# Independent control of cocontraction and reciprocal activity during goal-directed reaching in muscle space

**DOI:** 10.1038/s41598-020-79526-1

**Published:** 2020-12-18

**Authors:** Atsushi Takagi, Hiroyuki Kambara, Yasuharu Koike

**Affiliations:** 1 NTT Communication Science Laboratories, 3-1 Morinosato Wakamiya, Atsugi, Kanagawa 243-0198 Japan; 2 Institute of Innovative Research, Tokyo Institute of Technology, Yokohama, Japan; 3 Precursory Research for Embryonic Science and Technology (PRESTO), Japan Science and Technology Agency (JST), 4-1-8 Honcho, Kawaguchi, Saitama 332-0012 Japan

**Keywords:** Neuroscience, Motor control

## Abstract

The movement in a joint is facilitated by a pair of muscles that pull in opposite directions. The difference in the pair’s muscle force or reciprocal activity results in joint torque, while the overlapping muscle force or the cocontraction is related to the joint’s stiffness. Cocontraction knowingly adapts implicitly over a number of movements, but it is unclear whether the central nervous system can actively regulate cocontraction in a goal-directed manner in a short span of time. We developed a muscle interface where a cursor’s horizontal position was determined by the reciprocal activity of the shoulder flexion–extension muscle pair, while the vertical position was controlled by its cocontraction. Participants made goal-directed movements to single and via-point targets in the two-dimensional muscle space, learning to move the cursor along the shortest path. Simulations using an optimal control framework suggest that the reciprocal activity and the cocontraction may be controlled independently by the CNS, albeit at a rate orders of magnitude slower than the muscle’s maximal activation speed.

## Introduction

The human arm has several joints, wherein each joint is, at the very least, controlled by a pair of muscles that pull in opposing directions. Joint torque arises from the difference in the activity of a muscle pair, defined as the *reciprocal activity* (RA). A muscle pair can also pull against one another or *cocontract* by arbitrary amounts that result in zero net joint torque. We define cocontraction to be the *overlapping muscle force between the flexor and extensor muscles*.

The central nervous system (CNS) knowingly increases the arm’s cocontraction to accurately control the hand’s position during motion. Studies have reported an increase in the arm’s cocontraction at the end of a movement to stop the hand at a smaller target^1,2^, to move the hand accurately along a designated path^3,4^, and to stabilize the hand’s motion when it is deviated by unpredictable external forces^4–6^. The arm’s cocontraction also increases during impacts like when catching a falling object^7^ or striking a wall^8^. These increases in the cocontraction serve to augment the arm’s stiffness^9^, a mechanical property that restores the hand’s position to its unperturbed location without sensory delay. It is particularly useful when the brain cannot react fast enough to the unpredictable environment owing to the delays in proprioceptive or visual feedback.

The participants in the above-mentioned studies controlled their cocontraction to stabilize their arm to fulfil a task; the control of the cocontraction in itself was not the goal, and the participants received no feedback of their cocontraction to control it actively. Some studies have provided visual feedback of the arm’s cocontraction or stiffness to examine whether the CNS can actively control it. One study examined participants who exerted a static force in two dimensions while simultaneously controlling the arm’s stiffness^10^, but found that the coordination of the arm’s cocontraction between two joints was limited^11^ and could not be sustained^10^. Another study found that the cocontraction in the wrist could be controlled to a desired level set by the experimenter^12^. These studies only tested how the CNS could maintain the cocontraction at a designated constant value, and did not require the participants to modulate their cocontraction to different values in a short span of time. Thus, it is unclear to what extent the CNS can actively control the cocontraction to increase it or decrease it rapidly towards designated values.

The active control of cocontraction may be difficult for the CNS as most tasks require only a minimum threshold of cocontraction, and not a specific value per se^13^. Taking the example of catching a ball with the wrist, the CNS need only sustain a minimum threshold of cocontraction to keep the wrist’s position stable when the ball comes in contact with the hand. In such interaction tasks, there is no cocontraction target, but only a threshold to guarantee a successful catch. Additionally, the CNS may struggle to concurrently control reciprocal activity and cocontraction. When exerting a large RA to produce a big joint torque, the antagonist muscle also activates to stabilize the joint^14^, necessarily increasing the cocontraction. Thus, decreasing cocontraction while increasing RA should prove challenging for the CNS.

In light of this gap in the literature, this study investigates the proficiency of the CNS in actively regulating the cocontraction and the reciprocal activity simultaneously on short time scales, and to examine how the CNS executes the motor plan in muscle space. To test the CNS’ proficiency at regulating RA and cocontraction, we developed a muscle interface where a two dimensional cursor was controlled by shoulder cocontraction along the vertical axis and shoulder RA along the horizontal axis. Participants made goal-directed movements using our muscle interface. A computational simulation using an optimal control framework was used to assess whether the reciprocal activity and the cocontraction were controlled using a strategy to minimize the muscle activity needed to reach the targets in the muscle space.

## Materials and methods

### Experimental setup and protocol

8 right-handed male participants (23 ± 2 years old, all right-handed), who all gave written informed consent prior to the experiment, participated in the study, which was approved by the Ethical Review Board for Epidemiological Studies at the Tokyo Institute of Technology. All research was performed in accordance with the relevant guidelines and regulations.

**Figure 1:**
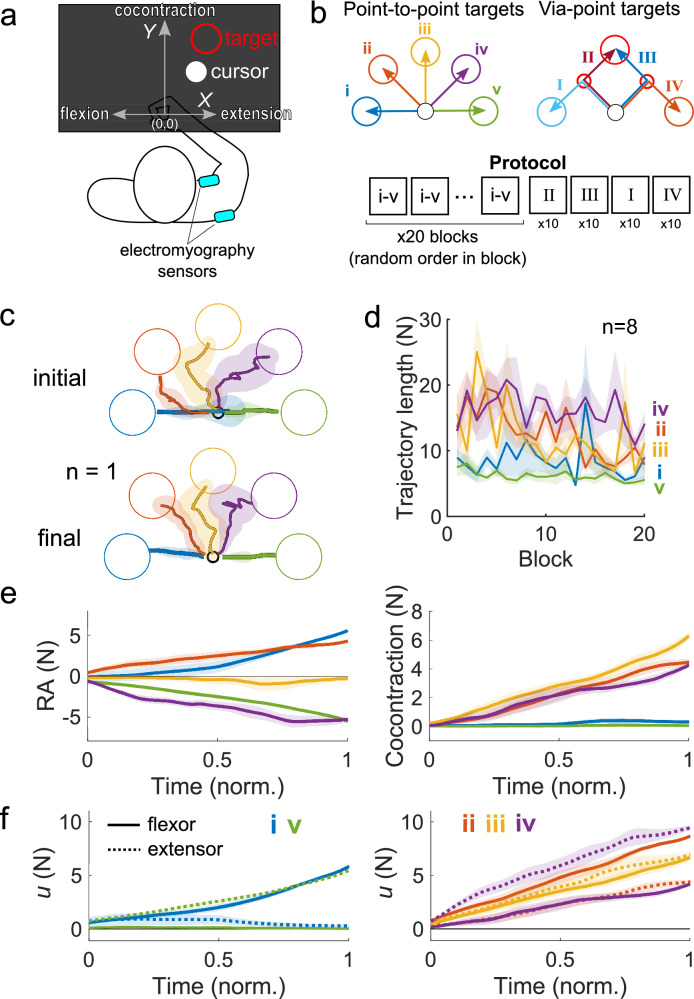
Experimental setup and protocol to test the muscle interface. (**a**) Participants held the handle of a robotic manipulandum whose position was fixed in front of the participant’s chest. Participants flexed and extended the shoulder muscle pair to move the cursor horizontally, and cocontracted the pair to move vertically. (**b**) Participants first executed point-to-point movements to one of five targets labelled (i) to (v), which were randomly presented in a block design. After the point-to-point reaching task, participants executed via-point movements along four paths, denoted (I)-(IV), for 10 repetitions. The ordering of the via-point targets was fixed. (**c**) The cursor’s trajectory from a representative participant from the initial and final phases of reaching the point-to-point targets. The length of the trajectory was noticeably shorter in the final phase for some targets. (**d**) The population mean trajectory length is plotted as a function of the block number (shaded area is one SEM). The trajectories to reach the flexion and extension targets (i) and (v) were the shortest. The length of the trajectory gradually decreased with training as participants learned to manipulate the cursor’s position using the muscle interface. (**e**) The time-series of the reciprocal activity and the cocontraction from the final phase of the point-to-point reaching task. A linear increase in the flexor and extensor activity was observed. Little RA was observed when reaching the cocontraction target (iii). (**f**) The time-series of the estimated force from the shoulder flexor and extensor muscles in the point-to-point task. Participants could activate each specific muscle by the correct magnitude to reach the target.

The participants were seated facing the KINARM planar robotic manipulandum from BKIN Technologies (Fig. 1a). Participants held onto the handle of the robotic interface, which was fixed in position by the robot’s motors, such that participants completed the task isometrically with the shoulder abducted at 45° and the elbow flexed such that the hand was 10 cm in front of the chest midline. An Edero Armon was used to support the arm’s weight. Visual feedback was provided on a monitor that was placed upside-down such that participants viewed a reflection of the monitor on a mirror placed above the hand that obscured it and the robot from view.

Two wireless EMG sensors (picoEMG, Cometa) were placed on the shoulder flexor (pectoralis major) and the shoulder extensor (posterior deltoid). The raw signals from the electromyography sensors were high-pass filtered at 10 Hz, rectified and low-pass filtered at 1 Hz. A second-order Butterworth filter was used for both the high-pass and low-pass filters.

In the point-to-point reaching task, participants had to reach and stop at one of either five targets {(i), (ii), (iii), (iv), (v)} (Fig. 1b). One block contained one instance of each point-to-point target. Participants were presented with targets drawn in random order from each block, experiencing twenty blocks in total. After the point-to-point reaching task, participants were then presented with ten repetitions of the via-point targets in the order of {(II), (III), (I), (IV)} (Fig. 1b), respectively. In total, this amounted to 140 reaching trials.

Participants were instructed to reach and stop inside the target circles. In the via-point task, they were instructed to pass through the via-point prior to reaching and stopping at the second, final target position. The radius of the final target was 3 cm on-screen while the via-point’s radius was 0.5 cm. The cursor’s velocity had to be below 10N/s and inside the target circle for the trial to succeed, which prevented participants from completing the movement by passing through the target at a high muscle activation rate.

### EMG normalization

Prior to the main experiment, the filtered EMG was normalized by asking participants to exert a 10N tangential force against the robot using the flexor or the extensor muscle alone 10 times per muscle. Visual feedback of the tangential force was provided continuously to the participant such that they exerted the force using the correct muscle. The data when the measured tangential force $\left\| F \right\|$ exceeded 1N was collated and regressed as a function of the filtered EMG activity of each muscle. For example, the force $\left\| F \right\|$ was regressed as a function of the filtered flexor muscle activity $m_{f}$

1 $$\left\| F \right\| = \alpha_{f} m_{f} + \beta_{f}$$

to identify the parameters of the fit $\left\{ {\alpha_{f} ,\beta_{f} } \right\}$ and $\left\{ {\alpha_{e} ,\beta_{e} } \right\}$ for the flexor and extensor muscles, respectively. The group mean values of the parameters were $\alpha_{f} = 998 \pm 12$, $\beta_{f} = 6.0 \pm 0.6$, $\alpha_{e} = 963 \pm 9$, $\beta_{e} = 5.0 \pm 0.8$. The variance explained by the fitted parameters was *R*^2^ = 0.81 ± 0.02.

In the main experiment, the force from each muscle was estimated using

2 $$\begin{aligned} \hat{F}_{f} \left( t \right) & = \alpha_{f} m_{f} \left( t \right) + \beta_{f} \\ \hat{F}_{e} \left( t \right) & = \alpha_{e} m_{e} \left( t \right) + \beta_{e} \\ \end{aligned}$$

where *t* is the time, $\hat{F}_{f} \left( t \right)$ is the estimated force from the shoulder flexor and $\hat{F}_{e} \left( t \right)$ is the estimated force from the shoulder extensor.

### Muscle interface

Participants controlled a cursor’s ($X\left( t \right)$*,*$Y\left( t \right)$) position on the monitor using shoulder reciprocal activity, defined as the difference between the flexor and extensor force $\hat{F}_{f} \left( t \right) - \hat{F}_{e} \left( t \right)$ where *t* is the time, and the shoulder cocontraction, defined as the minimum force of the flexor and extensor muscles ${\text{min}}\left( {\hat{F}_{f} \left( t \right),\hat{F}_{e} \left( t \right)} \right)$. The cursor’s position was given by

3 $$\left[ {\begin{array}{*{20}c} {X\left( t \right)} \\ {Y\left( t \right)} \\ \end{array} } \right] = 0.015\left[ {\begin{array}{*{20}c} {\hat{F}_{f} \left( t \right) - \hat{F}_{e} \left( t \right)} \\ {\min \left( {\hat{F}_{f} \left( t \right),\hat{F}_{e} \left( t \right)} \right)} \\ \end{array} } \right]$$

such that 1N of reciprocal activity or cocontraction displaced the cursor by 1.5 cm on the monitor.

Prior to the main experiment, we confirmed that participants could control the muscle interface by instructing them to push and pull the shoulder joint, and then asked them to cocontract the shoulder. This confirmed whether participants could move the cursor in the two-dimensional muscle space.

### Optimal control modelling of the cocontraction reaching task

The reaching task towards a force target in a two-dimensional muscle space is simulated using a damped mechanical system wherein the simulated reciprocal activity $u_{{{\text{RA}}}}$ and the simulated cocontraction $u_{{{\text{CC}}}}$ are under independent control with second-order muscle dynamics^15^. The muscle activation command passes through a spring-damper to yield the muscle force, mimicking the viscoelastic behavior of the muscles^16^. The flexor and extensor muscles were not simulated. $u_{{{\text{RA}}}}$ and $u_{{{\text{CC}}}}$ were scaled by 0.015 to yield the discretized position time-series $\left( {x_{i} ,y_{i} } \right)$ to compare it with the discretized data $\left( {X_{i} ,Y_{i} } \right)$. The control policy to determine the optimal simulated reciprocal activity and the simulated cocontraction are derived using a finite-horizon control framework^15^.

The state ${\mathbf{z}} = \left[ {u_{{{\text{RA}}}} ,\dot{u}_{{{\text{RA}}}} ,u_{{{\text{CC}}}} ,\dot{u}_{{{\text{CC}}}} } \right]^{T}$ evolves discretely with step size $dt = 0.01\,{\text{s}}$ and time index *k* according to damped mechanical dynamics

4 $${\mathbf{z}}_{k + 1} = {\mathbf{Az}}_{k} + {\mathbf{Bu}}_{k}$$

where $${\mathbf{u}}_{k} \equiv \left[ {\begin{array}{*{20}c} {\ddot{u}_{{{\text{RA}}}} } \\ {\ddot{u}_{{{\text{CC}}}} } \\ \end{array} } \right]$$ and

5 $${\mathbf{A}} = \left[ {\begin{array}{*{20}c} {{\mathbf{A}}_{1} } & {0_{2} } \\ {0_{2} } & {{\mathbf{A}}_{1} } \\ \end{array} } \right],\;{\mathbf{A}}_{1} = \left[ {\begin{array}{*{20}c} {1 - dt/\tau } & {dt/\tau } \\ 0 & {1 - dt/\tau } \\ \end{array} } \right], \;{\mathbf{B}} = \left[ {\begin{array}{*{20}c} {{\mathbf{B}}_{1} } & {0_{2,1} } \\ {0_{2,1} } & {{\mathbf{B}}_{1} } \\ \end{array} } \right],\;{\mathbf{B}}_{1} = \left[ {\begin{array}{*{20}c} 0 \\ {dt/\tau } \\ \end{array} } \right]$$

where $\tau$ is the time constant of the damped muscle dynamics, a free parameter whose influence is examined in greater detail in the next subsection. The controller regulates the rate of change of the reciprocal activity and the rate of change of the cocontraction.

The control command ${\mathbf{u}}_{k}$ minimizes the cost functional

6 $$J = \left( {{\mathbf{z}}_{{\varvec{N}}} - {\mathbf{Z}}_{N} } \right)^{T} {\mathbf{Q}}\left( {{\mathbf{z}}_{{\varvec{N}}} - {\mathbf{Z}}_{N} } \right) + \left( {{\mathbf{z}}_{{\varvec{v}}} - {\mathbf{Z}}_{{\varvec{v}}} } \right)^{T} {\mathbf{Q}}_{{\varvec{v}}} \left( {{\mathbf{z}}_{{\varvec{v}}} - {\mathbf{Z}}_{{\varvec{v}}} } \right) + \mathop \sum \limits_{i}^{N} {\mathbf{u}}_{i}^{T} {\mathbf{Ru}}_{i}$$

where *N* = 200 is the total length of the movement, chosen to be 2 s, equivalent to the group mean movement duration in all movements in all participants (2.0 ± 0.4 s), and where $v$ is the time index at which the via-point is reached in the simulation, which was identified from the data by choosing the time at which the trajectory was closest to the via-point. The target ${\mathbf{Z}}$ for the point-to-point movement was set to the population mean muscle force at the end of the movement for each target, ${\mathbf{Z}}_{N} = \frac{1}{0.015}\left[ {X_{N} ,0,Y_{N} ,0} \right]^{T}$, where $\left( {X_{N} ,Y_{N} } \right)$ is the final position of the cursor at the end of the movement. In the via-point simulation, the target was first set to the via-point target ${\mathbf{Z}}_{v} = \frac{1}{0.015}\left[ {X_{v} ,0,Y_{v} ,0} \right]^{T}$, where $\left( {X_{v} ,Y_{v} } \right)$ was the closest position that the cursor position passed by the via-point set in the experiment. The values of $\left( {X_{N} ,Y_{N} } \right)$ and $\left( {X_{v} ,Y_{v} } \right)$ were taken from the data to simulate each participant’s behavior in separate simulations. The cost functional is composed of the distance to the target at the end of the movement in the muscle space, the distance to the via-point at time $v$ and the running cost of the control inputs.

The control cost matrix is $${\mathbf{R}} = \left[ {\begin{array}{*{20}c} {10^{ - 4} } & 0 \\ 0 & {10^{ - 4} } \\ \end{array} } \right]$$ and the state cost matrix is $${\mathbf{Q}} = {\mathbf{Q}}_{v} = \left[ {\begin{array}{*{20}c} {{\mathbf{Q}}_{e} } & {0_{2} } \\ {0_{2} } & {{\mathbf{Q}}_{e} } \\ \end{array} } \right]$$ where $${\mathbf{Q}}_{e} = \left[ {\begin{array}{*{20}c} q & 0 \\ 0 & 0 \\ \end{array} } \right]$$. The weight of the state cost *q* is a free parameter whose influence on the simulated RA and cocontraction is examined in greater detail in the next subsection. To simulate the point-to-point movements, the via-point cost matrix ${\mathbf{Q}}_{v} = 0$.

The control command that minimizes the cost functional *J* is

7 $${\mathbf{u}}_{k} = - {\mathbf{L}}_{k} \left( {{\mathbf{z}}_{k} - {\mathbf{Z}}_{k} } \right)$$

where the target state ${\mathbf{Z}}_{k} = {\mathbf{Z}}_{N}$ is used to simulate the point-to-point movement. In the simulation of the via-point movements, ${\mathbf{Z}}_{k} = {\mathbf{Z}}_{v}$ when $k \le v$ and ${\mathbf{Z}}_{k} = {\mathbf{Z}}_{N}$ for $k> v$ . The control command ${\mathbf{u}}_{k}$ is computed backwards in time using

$${\mathbf{L}}_{k} = \left( {{\mathbf{R}} + {\mathbf{B}}^{T} {\mathbf{S}}_{k + 1} {\mathbf{B}}} \right)^{ - 1} {\mathbf{B}}^{T} {\mathbf{S}}_{k + 1} {\mathbf{A}}$$

8 $${\mathbf{S}}_{k} = {\mathbf{Q}}_{k} + {\mathbf{A}}^{T} {\mathbf{S}}_{k + 1} \left( {{\mathbf{A}} - {\mathbf{BL}}_{k} } \right)$$

which is initiated at the start with ${\mathbf{S}}_{N} = {\mathbf{Q}}_{N}$, and

9 $${\mathbf{Q}}_{k} = \left\{ {\begin{array}{*{20}c} {{\mathbf{Q}}_{N} ,\quad k = N} \\ {{\mathbf{Q}}_{v} ,\quad k = v} \\ {0,\quad \mathrm{otherwise}.} \\ \end{array} } \right.$$

Once the control policy is computed, the system is simulated forwards in time by iteratively computing Eq. (4).

### Effect of the free parameters τ and q

The simulation has two free parameters *τ* and *q* that influence the time-series of the simulated RA and simulated cocontraction in different ways. We systematically observed how the simulated muscle activity time-series changed with each parameter by modifying each parameter while the other was kept constant.

In the simulations of Fig. 3, the time constant was selected in the range $\tau = \left[ {0.16,10} \right]$ s in increments of 0.5 s while the state cost weight was kept constant at $q = 1000$. The state cost was selected from the set $q = \left\{ {10^{ - 6} ,10^{ - 5} ,10^{ - 4} ,10^{ - 3} ,10^{ - 2} ,0.1,1,10,100,10^{3} ,10^{4} ,10^{5} ,10^{6} ,10^{7} } \right\}$ while $\tau = 5.5$ s.

To identify the parameters that best explained the behavioral data, we simulated the RA and the cocontraction in the two-dimensional parameter space using the ranges of $\tau$ and *q* described above, and calculated the coefficient of determination $R^{2} = 1 - \frac{{SS_{{{\text{res}}}} }}{{SS_{{{\text{tot}}}} }}$ where $SS_{{{\text{res}}}} = \mathop \sum \limits_{i} \left( {X_{i} - x_{i} } \right)^{2} + \left( {Y_{i} - y_{i} } \right)^{2}$ and $SS_{{{\text{tot}}}} = \mathop \sum \limits_{i} \left( {X_{i} - \mathop X\limits } \right)^{2} + \left( {Y_{i} - \mathop Y\limits } \right)^{2}$, which quantified the variability in the data explained by the simulation. The values of $\tau$ and *q* that minimized $R^{2}$ were deemed the best set of parameters.

## Results

Motor learning of the muscle interface would be exhibited in how the cursor’s trajectory changed as a function of the block number. Figure 1c shows the cursor’s trajectory from a representative participant when reaching the five point-to-point targets denoted (i) to (v) (Fig. 1b), separated into the initial (first five blocks) and final (last five blocks) phase of the reaching task. Target (i) required solely shoulder flexion, and target (v) solely shoulder extension. Shoulder cocontraction alone was necessary to reach the target (iii), while a combination of reciprocal activity and cocontraction were required to reach targets (ii) and (iv). We noticed a reduction in the length of the cursor’s trajectory for select targets.

To quantify motor learning, we calculated the trajectory length in each trial, which is charted as a function of the block number (Fig. 1d). The trajectory length was computed in the initial and final phase of the task, and a two-way repeated measures ANOVA revealed a significant reduction in the trajectory length with the block number (F(1,7) = 21.3, *p* = 0.003) and a significant effect of the target (F(4,28) = 24.3, *p* < 0.001); the interaction between them was also significant (F(4,28) = 4.5, *p* = 0.006). Post-hoc comparisons using Tukey’s HSD revealed that the trajectory length when reaching targets (ii) (*p* < 0.001) and (iii) (*p* < 0.001) shortened with the block number. This learning effect was only observed for targets that required cocontraction, and not those where pure flexion (*p* = 0.99) or pure extension (*p* = 0.99) were needed. These results suggest that regulating either flexion or extension alone required little to no learning, while the concurrent control of reciprocal activity and cocontraction benefited from practice.

Figure 1e shows the group mean reciprocal activity and the cocontraction time-series in the final phase of the point-to-point reaching task (colors denote the different targets). For all targets, the increase in reciprocal activity and cocontraction was effectively linear. Similarly, the muscle activity of the shoulder flexor and extensor increased linearly with time (Fig. 1f), and their relative magnitude was carefully balanced to produce the RA and the cocontraction needed to reach each target.

**Figure 2:**
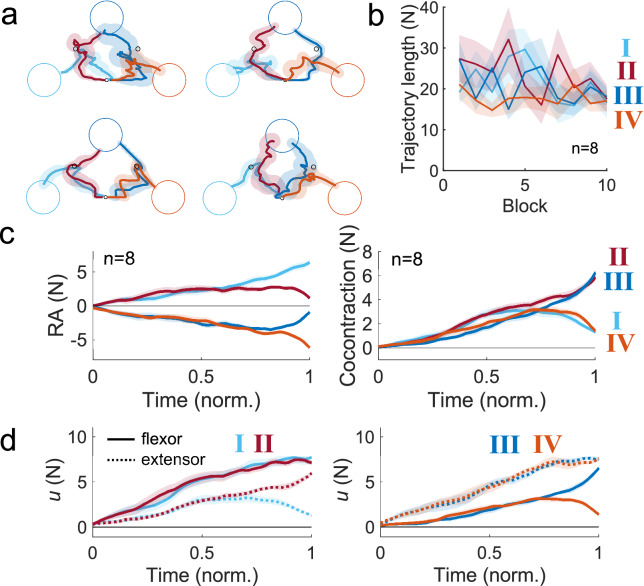
Participants successfully reached the via-point targets by modulating reciprocal activity and cocontraction. (**a**) The trajectory of four representative participants when reaching the four via-point targets is shown in the final phase of the task. Participants were successful at controlling the cursor’s position such that it passed through the via-point target prior to reaching the final target position. (**b**) The group mean length of the trajectories in reaching the four via-point targets as a function of the block number. The trajectory lengths were comparable between all via-point targets, implying that no specific combination of RA and cocontraction was particularly difficult for the nervous system to exert. (**c**) The time-series of the RA and the cocontraction in the via-point task. The increase in cocontraction was similar for all via-point targets, and only branched off in different directions approximately three-quarters of the way into the movement duration. (**d**) The time-series of the estimated shoulder flexor and extensor forces shows how the timely change in the RA occurred near the final half of the trial while the cocontraction was continually increasing.

Next, we examined the data from the via-point reaching task. Four representative participants’ cursor’s trajectories from the final phase of the via-point reaching task are charted in Fig. 2a. The cursor’s trajectory was more variable than in the point-to-point reaching task, possibly due to the additional complexity of having to change the reciprocal activity and the cocontraction halfway through the movement. The trajectory length was calculated as a function of the block number (Fig. 2b), and a two-way repeated measures ANOVA revealed no significant effect of the target type on the trajectory length (F(3,21) = 1.7, *p* = 0.2), but the trajectory did become significantly shorter with the block number (F(1,7) = 5.9, *p* < 0.046); the interaction between the factors was insignificant (F(3,21) = 1.2, *p* = 0.3). Thus, participants moved a shorter distance to fulfil the via-point reaching task, but the specific combination of increasing or decreasing RA and cocontraction did not influence the difficulty of the via-point task.

The time-series of the estimated muscle force from the shoulder flexor and extensor muscles (Fig. 2d) revealed the coordinated regulation of the RA and the cocontraction to move through the via-point and reach the final target (Fig. 2c). We checked whether the time index at which the cursor passed the via-point was dependent on the via-point target. A one-way repeated measures ANOVA with the via-point target as the factor found no significant effect on the via-point time index (F(3,21) = 2.6, *p* = 0.08), suggesting that the timing of passing the via-point was not dependent on the via-point target.

We developed a computational model of the reaching task based on an optimal control framework in the muscle space to determine whether the RA and the cocontraction were independently controlled by the CNS, and to examine how fast the CNS was able to modulate the activity in the muscle space to reach the targets. The simulated RA and the cocontraction were modelled as a damped system with independent control inputs that regulated the rate of change of the RA and the cocontraction. The flexor and the extensor activity were not simulated. The model has two free parameters that change the time-series of the simulated RA and cocontraction in different ways. The time constant *τ* determines the maximum rate at which the simulated RA and the cocontraction can be changed, corresponding to the cut-off frequency of a second-order filter. The weight on the state cost *q* penalizes the deviation in the simulated RA and cocontraction from the target values at the end of the movement (and at the time when passing through the via-point in the via-point task). We varied each of these parameters while keeping the other constant, and calculated the variance of the RA and the cocontraction explained by the simulation *R*^2^ (see Methods for details). For brevity, we only show the simulated RA in reaching the point-to-point target (i) when examining the effect of each free parameter on the time-series of the activity in the muscle space. However, all the point-to-point and via-point targets were simulated when calculating *R*^2^.

**Figure 3:**
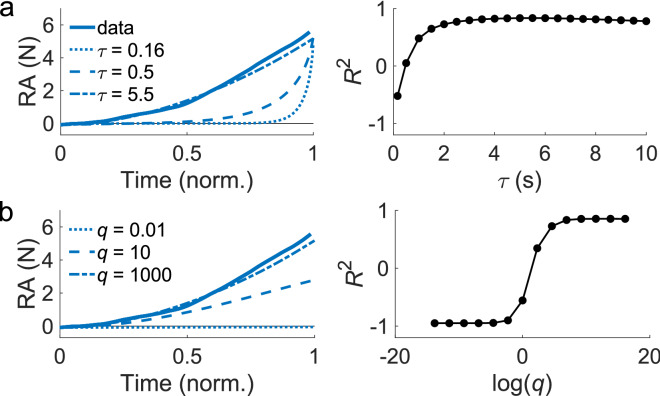
The values of the time constant *τ* and the weight on the state cost *q* changed the variability in the data explained by the model. (**a**) Time-series of the RA in the point-to-point task (pure flexion target) for different values of *τ*. The data is charted alongside for reference. The weight on the state cost $q = 1000$ was kept constant. *R*^2^ increased with larger *τ*, plateauing at $5.5$ s. (**b**) The weight on the state cost *q* was varied while $\tau = 5.5$ s was kept constant. *R*^2^ tended to increase with greater weight on the state cost *q*.

The time-series of the simulated RA for different values of the time constant are charted along with the empirical data for comparison (Fig. 3a). *R*^2^ increased with a greater time constant, but peaked at $\tau \approx 5.5$ s. A small value of $\tau$ enables the simulation to change the muscle activity rapidly such that the model only increases the RA at the end of the trial. In contrast, a high value of $\tau$ restricts the rate at which the RA increases, resulting in a gradual increase that resembles the behavior of our participants. Our participants likely could not control the RA and the cocontraction faster than $\tau = 5.5$ s, which corresponds to a cut-off frequency of 0.03 Hz of the damped second-order muscle dynamics used in our model.

The time-series of the simulated RA for different values of the weight on the state cost *q* is plotted in Fig. 3b. The simulation was closer to the data with a higher value of *q*. When *q* is too small the controller is unwilling to increase the muscle activity to minimize the difference between the simulated and the target RA at the end of the trial due to the running cost of the control inputs.

**Figure 4:**
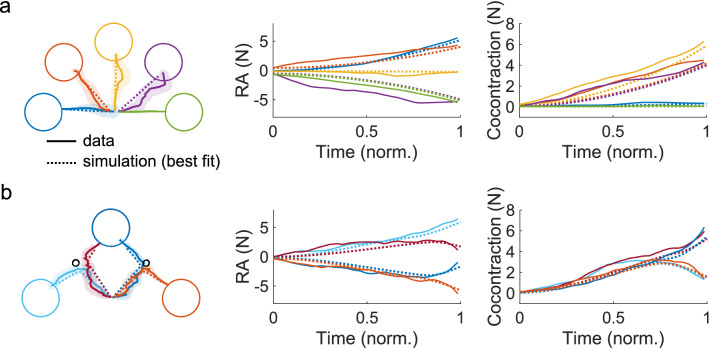
The model with the parameters *τ* and *q* that best fit the empirical data could capture the variability in the group mean RA and cocontraction with *R*^2^ = 0.87. (**a**) The group mean trajectories for the point-to-point targets from the data (solid trace) and the simulation (dashed trace) are shown. The time-series of the RA and the cocontraction from the data and the simulation resembled one another. (**b**) The group mean trajectories from the via-point reaching task from the data and the simulation are plotted. The time-series of the RA and the cocontraction from the simulation resembled the participants’ behavior.

We then conducted simulations in the 2D parameter space, and found that the values of the free parameters that yielded the greatest $R^{2} = 0.87$ was $\tau = 5.5$ s and $q = 1000$. The trajectories in the RA and cocontraction muscle space from the empirical data and this best simulation are plotted in Fig. 4a,b for the point-to-point and the via-point tasks, respectively. The time-series of the simulated RA and the simulated cocontraction resembled those from the data.

## Discussion

To examine the ability of the CNS to concurrently control the cocontraction and the reciprocal activity, we developed an interface that mapped a cursor’s movement in two dimensions where the horizontal axis was controlled by RA and the vertical axis was controlled using cocontraction. Muscle activity alone was employed to control the cursor’s position, which was used to reach point targets in the two-dimensional muscle space. The length of the cursor’s trajectory decreased with the block number in the point-to-point reaching task, suggesting that as participants grew familiar with the interface, they gradually decreased the distance travelled by the cursor in reaching equidistant targets. No decrease was observed when solely flexion or extension sufficed to reach the target, indicating that the motor learning occurred primarily in controlling the shoulder cocontraction.

By modifying the time constant of the muscle activity in the simulation, we determined the rate at which the RA and the cocontraction was controlled by our participants. The muscles in the arm can be modelled as a mechanically damped system with a cut-off frequency of 4Hz^15,17^, or a time constant of 0.04 s. The fastest our interface could be controlled was at 1 Hz due to the low-pass filtering of the EMG when estimating the muscle forces. Our results were best explained by a time constant of 5.5 s, corresponding to a cut-off frequency of 0.03 Hz, significantly lower than the maximum rate of 1 Hz which was possible using our interface. This is a limitation of our model, as we had to tune the time constant determining the muscle dynamics, rather than taking the 1 Hz value used in the actual muscle interface.

The large time constant could be due to a number of factors. As the participants rely purely on visual feedback to control the cursor’s position in the muscle space, this could have limited the rate at which the RA and the cocontraction could be controlled. The control in the muscle space could also be slower due to the normalization of the filtered muscle activity, which was linearized prior to the main experiment. The linearization can break down with greater muscle activity^16^ and fatigue^18^, which could have reduced the participants’ ability to control their RA and the cocontraction. Furthermore, our muscle model is limited at explaining the data as it did not consider signal-dependent noise^19^ when controlling the RA and the cocontraction. Signal-dependent noise can punish the controller to avoid rapid changes in the muscle space, and may also lower the effective time constant of the muscle activity needed to explain the data^20^.

To our knowledge, our task is unique in exploring the active control of cocontraction. Related studies in the literature asked participants to maintain either a fixed value of stiffness or cocontraction^11,12^, or to maintain a constant joint torque while modulating the stiffness^21^. In contrast, our task required simultaneous changes in both RA and cocontraction to reach a target in the muscle space, resembling two-dimensional reaching tasks that have been extensively examined in the field of motor control^22^. Due to this resemblance with previous studies on reaching movements, we used an optimal control framework commonly used in reaching studies to simulate the control of the RA and the cocontraction^15^. The control of the RA and the control of the cocontraction must necessarily be independent in the model to explain the data. Our participants were able to reach the point-to-point targets that required only the RA, suppressing cocontraction, and could also increase the cocontraction with nearly null RA. Our model suggests that the participants may have minimized a cost functional used commonly in the motor control literature, wherein the deviation from the target’s position at the end of the movement and the running cost of effort are minimized.

The ability to modulate the RA and the cocontraction independently of one another raises the interesting possibility of an additional control input for human–computer interfaces. The cocontraction in the joint could be used as an auxiliary control input to augment human motor function e.g., using the shoulder cocontraction to rotate a platform during soldering. However, the muscle interface in its current form may be too slow to meaningfully control a device. To be viable as a novel human–computer interface, further innovation is needed to boost the rate at which the muscle interface can be controlled.
